# The Role of Glucose-6-phosphate Dehydrogenase in the Wine Yeast *Hanseniaspora uvarum*

**DOI:** 10.3390/ijms25042395

**Published:** 2024-02-18

**Authors:** Jürgen J. Heinisch, Andrea Murra, Lucía Fernández Murillo, Hans-Peter Schmitz

**Affiliations:** AG Genetik, Fachbereich Biologie/Chemie, Universität Osnabrück, Barbarastr. 11, D-49076 Osnabrück, Germany; amurra@uni-osnabrueck.de (A.M.); lucfernandez@uni-osnabrueck.de (L.F.M.); hans-peter.schmitz@uni-osnabrueck.de (H.-P.S.)

**Keywords:** *ZWF1*, pentose phosphate pathway, sulfite resistance, oxidative stress, human G6PDH

## Abstract

*Hanseniaspora uvarum* is the predominant yeast species in the majority of wine fermentations, which has only recently become amenable to directed genetic manipulation. The genetics and metabolism of *H. uvarum* have been poorly studied as compared to other yeasts of biotechnological importance. This work describes the construction and characterization of homozygous deletion mutants in the *HuZWF1* gene, encoding glucose-6-phosphate dehydrogenase (G6PDH), which provides the entrance into the oxidative part of the pentose phosphate pathway (PPP) and serves as a major source of NADPH for anabolic reactions and oxidative stress response. *Huzwf1* deletion mutants grow more slowly on glucose medium than wild-type and are hypersensitive both to hydrogen peroxide and potassium bisulfite, indicating that G6PDH activity is required to cope with these stresses. The mutant also requires methionine for growth. Enzyme activity can be restored by the expression of heterologous G6PDH genes from other yeasts and humans under the control of a strong endogenous promoter. These findings provide the basis for a better adaptation of *H. uvarum* to conditions used in wine fermentations, as well as its use for other biotechnological purposes and as an expression organism for studying G6PDH functions in patients with hemolytic anemia.

## 1. Introduction

Glucose-6-phosphate dehydrogenase (G6PDH; EC 1.1.1.49) catalyzes the first step in the oxidative part of the pentose phosphate pathway (PPP, in which glucose-6-phosphate is converted into 6-phosphoglucono lactone. This enables the production of pentose phosphates required as precursors for the synthesis of nucleic acid, cofactors, and histidine, as well as erythrose-4-phosphate in the non-oxidative part of the pathway for aromatic amino acid biosynthesis [1,2]. In addition, the G6PDH reaction is the primary source of NADPH in many cells and tissues, providing the reducing power to drive lipid and fatty acid synthesis and for the detoxification of reactive oxygen species (ROS). This antioxidant activity explains the importance of the G6PDH reaction in cancerogenesis [3,4] and neurodegenerative diseases [5]. NADPH is also required for contractility in cardiomyocytes and is thus important for proper heart function [6]. With respect to human health, G6PDH is of utmost importance in erythrocytes, being the exclusive source of NADPH [7]. In fact, malfunctions of the enzyme are the leading cause of hemolytic anemia, with some 217 mutations described for the encoding gene [8]. Predisposition for this enzymopathic condition may affect as many as 500 million people worldwide, most being asymptomatic unless exposed to certain diets or drugs [9]. In addition, increased enzyme activities have been detected in a variety of human tumors [10].

Mutants in the *ZWF1* gene (for the German “Zwischenferment” derived from biochemical studies early in the last century [11]) were first obtained by suppression of the glucose-negative phenotype of a 6-phosphogluconate dehydrogenase mutant [12]. Later on, deletion mutants constructed after gene cloning were found to be hypersensitive towards oxidative stress agents and to require methionine for growth, all attributed to the reduced provision of NADPH for detoxification or biosynthesis, respectively [13,14,15]. Vice versa, oxidative stress inactivates the glycolytic enzymes glyceraldehyde-3-phosphate dehydrogenase and triosephosphate isomerase, which results in enhancing the flux through G6PDH and NADPH production [16]. The latter has been attributed to the recycling of glucose-6-phosphate by means of the non-oxidative part of the PPP rather than metabolite accumulation caused by the block in glycolysis [17]. While a *zwf1* deletion has little effect on the growth of glucose in the genetic background of some common laboratory strains of *S. cerevisiae* [15,18], it grows poorly under standard conditions in a strain more closely related to industrial baker’s yeast compared to its isogenic wild-type [19]. These defects can be complemented by introducing genes encoding heterologous G6PDH enzymes from humans and plants so that the deletion strain has been proposed as an expression organism for production and biochemical studies of the heterologous enzymes [19].

While *S. cerevisiae* has been employed as a general model system in biochemistry and molecular genetics, it is specialized in alcoholic fermentation and has been selected for this purpose by mankind for thousands of years [20]. In fact, it reduces its respiratory capacity in the presence of high medium sugar concentrations, independent of the availability of oxygen (the so-called Crabtree effect), and thus does not display the Pasteur effect (i.e., switching to respiratory metabolism when oxygen is available, [21]). The PPP is, therefore, of greater importance in Crabtree-negative yeasts, which require respiration for efficient energy production instead of relying on glycolytic capacity [1]. Amongst these yeasts, *Kluyveromyces lactis* acquired a model status, as it has not undergone a whole genome duplication and can be manipulated by similar techniques of classical and molecular genetics as used for *S. cerevisiae* [22,23]. Evidence for a more prominent role of the PPP in sugar metabolism by this yeast was provided by the fact that glycolytic mutants, such as *Klpgi1* or *Klpfk1 Klpfk2* deletions, still grew on glucose as a sole carbon source, in contrast to its *S. cerevisiae* counterparts [24,25]. The notion of increased flux through the PPP was strongly supported by an additional lack of transaldolase in the respective double (*Klpgi1 Kltal1*) and triple (*Klpfk1 Klpfk2 Kltal1*) deletions, which failed to grow on glucose [26]. A lack of G6PDH activity in *Klzwf1* deletions leads to reduced biomass production on different carbon sources as compared to the wild-type [27]. The active enzyme in *K. lactis* is a homotetramer, which is allosterically regulated by NADPH, upon whose binding it dissociates into dimers [28].

*Hanseniaspora uvarum* is another Crabtree-negative yeast which draws growing attention for its biotechnological potential [29]. Although it is found on grapes in vineyards worldwide and frequently dominates the yeast population in the first hours of wine fermentation [30,31,32], its genetics and physiology have been poorly studied at the molecular level. A first basis for such studies was provided by sequencing the genome and its annotation, accompanied by determinations of glycolytic enzyme activities [33]. The latter work attributed the lower fermentative capacity of *H. uvarum* as compared to *S. cerevisiae* to a reduced pyruvate kinase activity as the final step of glycolysis. Based on the genome sequence methods for the introduction of DNA and the construction of deletion mutants have been successfully implemented only recently [34,35]. The construction of autonomously replicating plasmids and homozygous auxotrophic deletion mutants in the diploid-type strain (i.e., *Huade2*, *Huhis3*, *Huleu2*, and *Huura3*) provided a set of essential tools for future genetic manipulations of this yeast [36].

Here, we applied this toolset for the construction and physiological characterization of deletion mutants in the *HuZWF1* gene. These were then employed as a host for the expression of heterologous genes encoding G6PDH from other yeasts, as well as a human isoform.

## 2. Results

### 2.1. Homozygous Huzwf1 Deletion Mutants Lack Glucose-6-phosphate Dehydrogenase Activity

**Figure 1:**
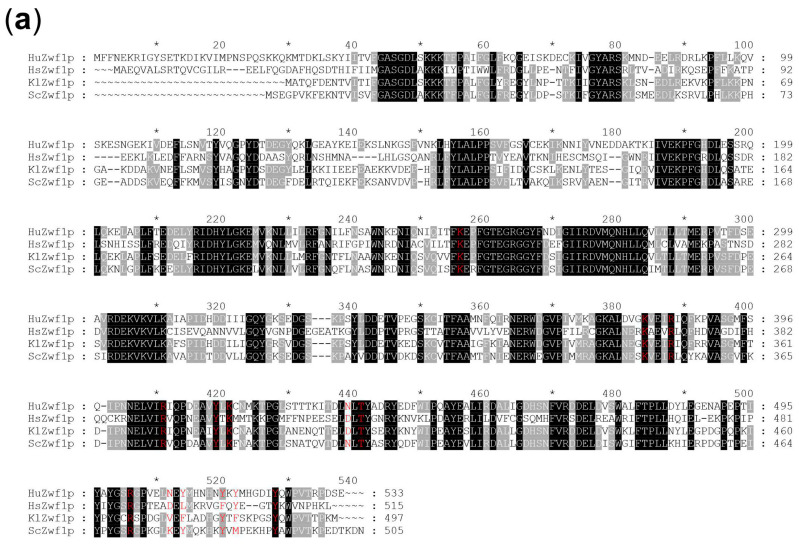

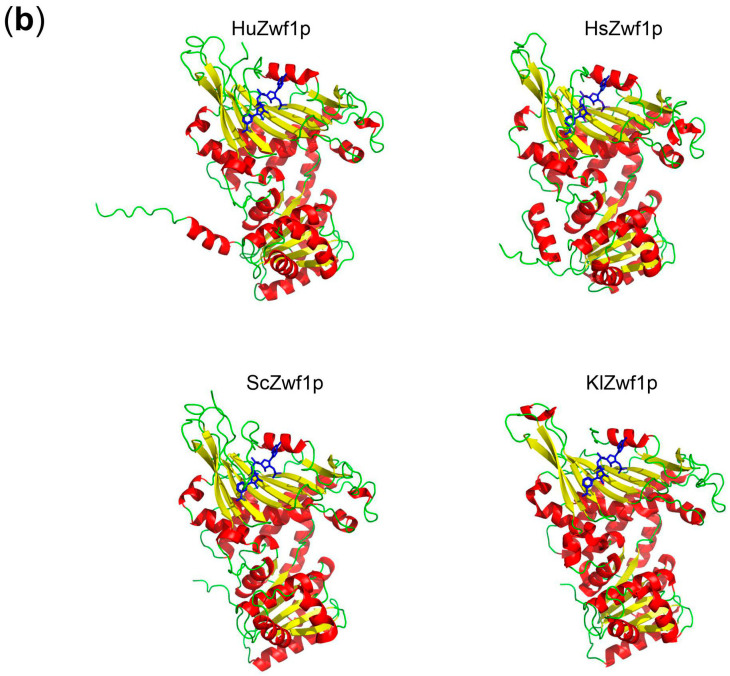
In silico analyses of HuZwf1. (**a**) Alignment of the deduced amino acid sequences of HuZwf1p (Uniprot accession number A0A1E5S1A9) with the G6PDHs from a human isoform (designated here as HsZwf1p; Uniprot accession number P11413), baker’s yeast (ScZwf1p; Uniprot accession number P11412), and (KlZwf1p; Uniprot accession number P48828). Identical amino acid residues are shown with a black background and conserved residues with a light gray background. Amino acid residues known to participate in glucose-6-phosphate binding are highlighted in red letters. Numbering of amino acids is in intervals of twenty, with an “*” after ten residues in between. (**b**) Proposed structures of HuZwf1p compared to the human G6PDH obtained with the AlphaFold 2 program ([37,38]). Alpha-helical domains are shown in red and ß-sheets as yellow arrows. The cofactor (NADP^+^) in its binding pocket is indicated in blue. The latter is located close to the site of glucose-6-phosphate binding at the interfaces of monomers in the tetrameric enzyme.

Searching the annotated genome sequence of *Hanseniaspora uvarum* for homologies to glucose-6-phosphate dehydrogenases (G6PDH), we identified one candidate gene to encode the enzyme, further designated as *HuZWF1*. The deduced amino acid sequence is comprised of 533 residues and was aligned with the homologues from *Saccharomyces cerevisiae* (ScZwf1p), *Kluyveromyces lactis* (KlZwf1p), and a human isoform (HsZwf1p; Figure 1a, see legend for accession numbers), sharing 63%, 67%, and 45% overall identity with HuZwf1, respectively. Domains known to bind glucose, or the nucleotide cofactor, were more strongly conserved, indicating that *HuZWF1* indeed encodes a functional homologue. This notion was further supported by the modelling of the three-dimensional structure, which was highly similar to human G6PDH and the enzymes from the two other yeast species for the core structure (Figure 1b). Interestingly, HuZwf1p shares an additional N-terminal helix with the human isoform, which is not present in the other yeast enzymes.

**Figure 2:**
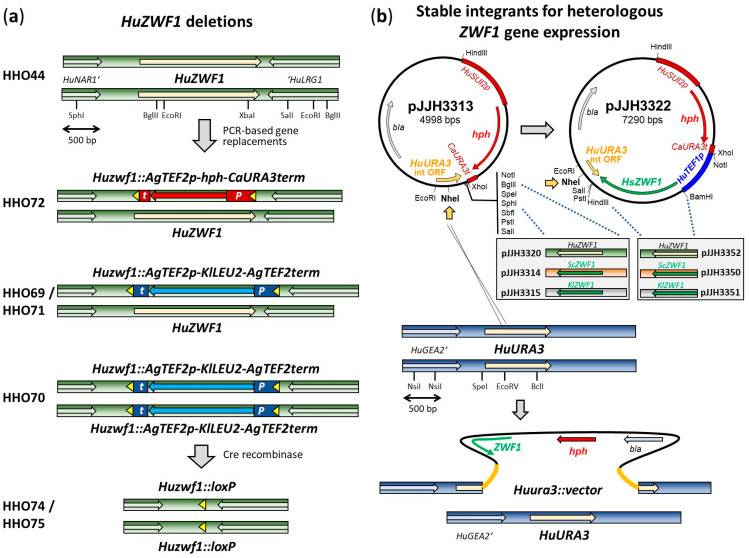
Genomic constitution of manipulated *H. uvarum* strains. Bars of different colors represent the genomic loci. Open reading frames are depicted as colored arrows. Promoter (P) and terminator (t) sequences are shown as boxes. (**a**) *HuZWF1* locus of strains carrying deletions of one or two *HuZWF1* alleles. The diploid recipient strain HHO44 also carries a homozygous deletion at the *HuLEU2* locus. Names of the resulting strains are shown at the left. Yellow triangles represent *loxP* sites (not shown to scale) as target sequences for the Cre recombinase. Selected restriction sites are indicated in the upper part for orientation. *HuNAR1′* and ‘*HuLRG1* designate the ends of the flanking open reading frames from genes homologous to the *S. cerevisae* genome. (**b**) Construction of stable integrants for expression of heterologous *ZWF1* genes in HHO75. For expression of heterologous genes under their native promoter plasmid, pJJH3313 was constructed as described in materials and methods (Section 4.2). A unique NheI site not present in the genomic sequence was introduced into the internal fragment of the *HuURA3* open reading frame (*HuURA3* int ORF) and used to direct integration by homologous recombination. The gray box in the middle lists the genes inserted into the polylinker region and the names of the resulting constructs (not drawn to scale). For the expression of the heterologous genes under the control of the strong *HuTEF1* promoter, pJJH3322 was used as a recipient vector (upper right corner). The *HsZWF1* gene was replaced by the fragments indicated in the gray box below the plasmid map, with names of the resulting constructs indicated (not drawn to scale). Integration into the *HuURA3* locus resulted in the disruption of one of the alleles, as shown at the bottom. *HuGEA2′* depicts the end of the open reading frame of a gene homologous to *S. cerevisiae*. Scale bars in figure (**a**,**b**) apply to all linear maps, unless indicated otherwise.

In order to provide experimental proof for this enzyme function, we decided to delete the encoding gene from strain HHO44 (*Huleu2*/*Huleu2*). Since this strain is diploid, a strategy to consecutively substitute the two alleles with different marker cassettes was originally employed, as exercised previously for the auxotrophic markers (Figure 2a; [36]). However, in the course of verification by PCR, two clones turned out to carry the homozygous deletion of both alleles with the *KlLEU2* cassette in a single transformation of HHO44, which were further employed.

The resulting strains were then grown in rich medium into a logarithmic phase and harvested for the preparation of crude extracts. Specific G6PDH activities were determined and are listed in Table 1 (upper part).

**Table 1:** Specific G6PDH activities.

Strain	Relevant *ZWF1* Alleles	mU/mg Protein
HHO1	Wild-type	252 ± 11
HHO44	Wild-type	257 ± 4
HHO69	*Huzwf1Δ/HuZWF1*	132 ± 16
HHO72	*Huzwf1Δ/HuZWF1*	123 ± 13
HHO70	*Huzwf1Δ/Huzwf1Δ*	b.d.
HHO75	*Huzwf1Δ/Huzwf1Δ*	b.d.
HHO75/pJJH3313	*Huzwf1Δ/Huzwf1Δ Huura3::vector/HuURA3*	b.d.
HHO75/pJJH3320	*Huzwf1Δ/Huzwf1Δ Huura3::HuZWF1/HuURA3*	168 ± 2
HHO75/pJJH3314	*Huzwf1Δ/Huzwf1Δ Huura3::ScZWF1/HuURA3*	11 ± 1
HHO75/pJJH3315	*Huzwf1Δ/Huzwf1Δ Huura3::KlZWF1/HuURA3*	8 ± 1
HHO75/pJJH3352	*Huzwf1Δ/Huzwf1Δ Huura3::TEF1p-HuZWF1/HuURA3*	651 ± 27
HHO75/pJJH3350	*Huzwf1Δ/Huzwf1Δ Huura3::TEF1p-ScZWF1/HuURA3*	349 ± 27
HHO75/pJJH3351	*Huzwf1Δ/Huzwf1Δ Huura3::TEF1p-KlZWF1/HuURA3*	175 ± 5
HHO75/pJJH3322	*Huzwf1Δ/Huzwf1Δ Huura3::TEF1p-HsZWF1/HuURA3*	98 ± 12

Given are the mean specific activities of at least three biological replicates, each with three technical replicates (with the exception of the second wild-type, HHO44, and the homozygous deletions HHO70 and HHO75, for which biological duplicates were examined). Species designations for *ZWF1* genes are *Hu = Hanseniaspora uvarum, Sc = Saccharomyces cerevisiae, Kl = Kluyveromyces lactis, and Hs = Homo sapiens*. For complete genotypes and constitution of the deletion alleles, consult Table 2. b.d. = below detection (<2 mU/mg protein).

**Table 2:** *Hanseniaspora uvarum* strains used in this work.

Strain	Genotype	Reference
HHO1	Wild-type	[36]
HHO44	*Huleu2::loxP/Huleu2::loxP*	[36]
HHO68	*Huleu2::loxP/Huleu2::loxP Huzwf1::KlLEU2/Huzwf1::KlLEU2*	this work
HHO69	*Huleu2::loxP/Huleu2::loxP Huzwf1::KlLEU2/HuZWF1*	this work
HHO70	*Huleu2::loxP/Huleu2::loxP Huzwf1:: KlLEU2/Huzwf1:: KlLEU2*	this work
HHO71	*Huleu2::loxP/Huleu2::loxP Huzwf1:: KlLEU2/HuZWF1*	this work
HHO72	*Huleu2::loxP/Huleu2::loxP Huzwf1::hph/HuZWF1*	this work
HHO74	*Huleu2::loxP/Huleu2::loxP Huzwf1::loxP/Huzwf1::loxP*	this work
HHO75	*Huleu2::loxP/Huleu2::loxP Huzwf1::loxP/Huzwf1::loxP*	this work
HHO75/pJJH3313	*Huleu2::loxP/Huleu2::loxP Huzwf1::loxP/Huzwf1::loxP Huura3::hph/HuURA3*	this work
HHO75/pJJH3320	*Huleu2::loxP/Huleu2::loxP Huzwf1::loxP/Huzwf1::loxP Huura3::HuZWF1-hph/HuURA3*	this work
HHO75/pJJH3314	*Huleu2::loxP/Huleu2::loxP Huzwf1::loxP/Huzwf1::loxP Huura3::ScZWF1-hph/HuURA3*	this work
HHO75/pJJH3315	*Huleu2::loxP/Huleu2::loxP Huzwf1::loxP/Huzwf1::loxP Huura3::KlZWF1-hph/HuURA3*	this work
HHO75/pJJH3352	*Huleu2::loxP/Huleu2::loxP Huzwf1::loxP/Huzwf1::loxP Huura3::TEF1p-HuZWF1-hph/HuURA3*	this work
HHO75/pJJH3350	*Huleu2::loxP/Huleu2::loxP Huzwf1::loxP/Huzwf1::loxP Huura3::TEF1p-ScZWF1-hph/HuURA3*	this work
HHO75/pJJH3351	*Huleu2::loxP/Huleu2::loxP Huzwf1::loxP/Huzwf1::loxP Huura3::TEF1p-KlZWF1-hph/HuURA3*	this work
HHO75/pJJH3322	*Huleu2::loxP/Huleu2::loxP Huzwf1::loxP/Huzwf1::loxP Huura3::TEF1p-HsZWF1-hph/HuURA3*	this work

*hph* designates the gene conferring resistance to hygromycin B; HHO74 and HHO75 are two clones derived from HHO70 after elimination of the *KlLEU2* marker by Cre-induced recombination.

As expected from retaining one intact gene copy, the heterozygous deletion mutants (HHO69 and HHO72) displayed approximately half of the specific G6PDH activity as the wild-type controls (HHO1 and HHO44), whereas the homozygous deletions (HHO74 and HHO75) showed no detectable activity. This clearly demonstrates that *HuZWF1* indeed encodes G6PDH, both alleles encode functional subunits, and that the *H. uvarum* genome carries no further genes encoding a functional enzyme. This notion was further supported by re-introducing a copy of the wild-type *HuZWF1* gene into the homozygous deletion strain (HHO75/pJJH3320), which was integrated at the *HuURA3* locus by disrupting one of the alleles, which restored enzyme activity to half the level of the wild-type controls (Table 1, lower part).

### 2.2. Heterologous Genes Restore G6PDH Activity in Huzwf1 Deletion

Given the striking similarities in G6PDH enzyme structures depicted in Figure 1b and the importance of the enzyme in human health, we wondered whether *H. uvarum* could serve as an expression organism to study enzymatic parameters of genes expressed from patients or simply for comparative studies of G6PDHs from different sources. Therefore, heterologous genes from humans and the two model yeasts *S. cerevisiae* and *K. lactis* were cloned into an integrative vector and stably integrated into the genome of HHO75 (*Huzwf1Δ/Huzwf1Δ*), disrupting one of the two *HuURA3* alleles in the process (Figure 2b). Cells were logarithmically grown in rich medium and harvested for the preparation of crude extracts and the determination of specific G6PDH activities (Table 1, lower part). As expected, integration of the empty vector (pJJH3313) did not restore enzymatic activity, but the integration of a single copy of the *HuZWF1* gene under the control of its native promoter yielded half the specific activity of the wild-type control. Strains carrying either the gene from *S. cerevisiae* (*ScZWF1*) or that from *K. lactis* (*KlZWF1*) under their native promoters yielded only very low specific G6PDH activities, indicating that these promoters are poorly recognized for efficient gene expression in *H. uvarum*. Consequently, both genes were placed under the control of the strong native *HuTEF1* promoter. Specific G6PDH activities of approximately 350 mU/mg protein (*HuTEF1p-ScZWF1*) and 175 mU/mg protein (*HuTEF1p-KlZWF1*) in crude extracts from these strains demonstrated functional expression in the broad range of the native HuZwf1p enzyme in wild-type strains. Placing the native gene under the strong promoter (*HuTEF1p-HuZWF1*) yielded the highest specific activities (approximately 650 mU/mg protein), i.e., triplicating the value of the wild-type control. Finally, the strain expressing the human gene (*HuTEF1p-HsZWF1*) still displayed activities of approximately 100 mU/mg protein. These findings provide proof-of-principle that *H. uvarum* is a suitable host for heterologous gene expression.

### 2.3. Lack of HuZwf1 Confers Methionine Auxotrophy and Hypersensitivity towards Hydrogen Peroxide and Sulfite, Whereas Heterologous Expression of a Sulfite Transporter Increases Resistance

**Figure 3:**
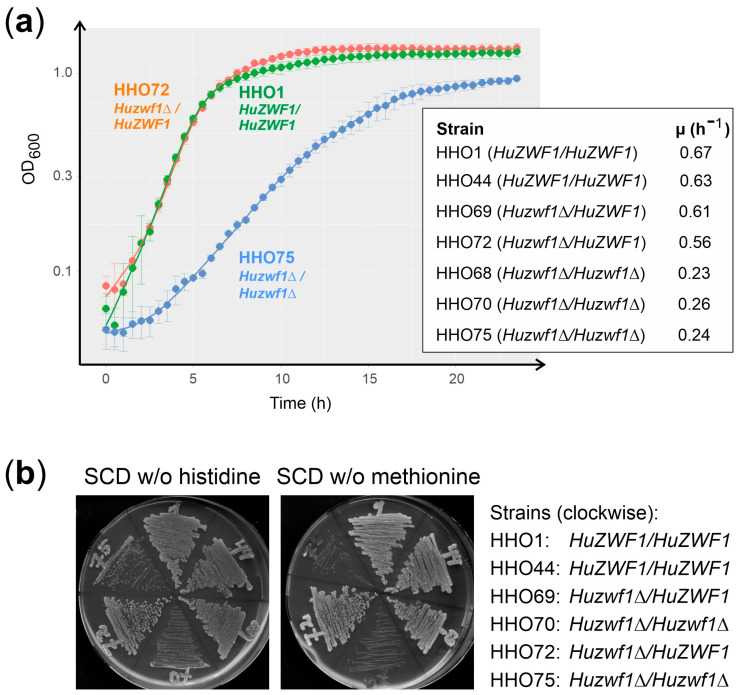
Strains lacking *HuZWF1* have a reduced growth rate and require methionine. (**a**) Growth curves were recorded with a Varioscan Lux plate reader at 28 °C as detailed in materials and methods (Section 4.3). Error bars at each point indicate the standard deviations of three technical replicates for each strain. A representative example of each genetic constitution is depicted in the growth curves. Note that the *Y*-axis is a logarithmic scale. The table lists the maximal growth rates (µ) determined for the strains and relevant genotypes as indicated. (**b**) Cells from overnight cultures in rich medium (YEPD) were streaked out onto the media indicated and incubated for 3 days at 25 °C. Numbers on the plates refer to the HHO nomenclature of the strains as indicated at the right, also showing the relevant genotypes (with HHO1 as the wild-type control).

As we noticed that the homozygous *Huzwf1* deletion mutants produced smaller colonies when streaked out on plates with glucose as a carbon source, we proceeded by recording their growth curves (Figure 3a).

The reducing power in the form of NADPH generated by the G6PDH reaction is required for methionine biosynthesis and to combat oxidative stress both in *S. cerevisiae* and *K. lactis* [15,27]. The *Huzwf1* deletion strains were therefore tested for their ability to grow on synthetic medium lacking methionine. As evident from Figure 3b, the homozygous deletion strains display severe growth retardation on synthetic medium supplemented with methionine as compared to the wild-type controls and the heterozygous deletion strains still carrying one functional gene copy (note that histidine was omitted from the control plates to demonstrate that the strains did not display a general growth defect in the absence of amino acids). However, the homozygous deletions do not grow when methionine is omitted, in contrast to the other strains. We concluded that while sufficient NADPH for methionine synthesis can be produced by strains carrying one gene copy of *HuZWF1*, a complete lack of enzyme activity (in strains HHO70 and HHO75) renders them auxotrophic for this amino acid.

**Figure 4:**
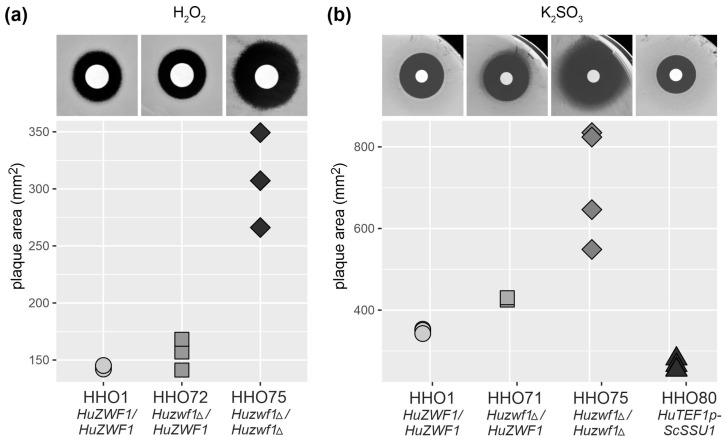
Strains lacking *HuZWF1* are hypersensitive towards oxidative stress and sulfite. In the upper row, representative examples of the halo assays are shown for the strains indicated at the bottom. Quantitative analyses of at least two biological replicates for each strain with the area of inhibition are shown in the lower part. (**a**) Resistance towards hydrogen peroxide (2.8 mM). (**b**) Resistance towards potassium bisulfite (3.6 M).

In order to test the sensitivity of the strains towards oxidative stress and sulfite, we employed growth inhibition halo assays (Figure 4). To assess the reaction towards oxidative stress, sterile filter discs soaked with 10 µL of different hydrogen peroxide concentrations were placed on a lawn of cells to determine the zones of inhibition after overnight incubation at 28 °C (with a concentration of 2.8 mM shown in the upper part of Figure 4a).

Strains with a heterozygous deletion (*Huzwf1Δ/HuZWF1*) displayed a similar sensitivity to the wild-type, as indicated by the respective halo areas, whereas homozygous deletions (*Huzwf1Δ/Huzwf1Δ*) were much more sensitive. We concluded that *H. uvarum*, like other yeasts, requires G6PDH activity to efficiently cope with oxidative stress.

Wine production commonly involves treatment with sulfite as an antioxidant and for microbial stabilization. Although *H. uvarum* frequently predominates the yeast population at the beginning of wine fermentations, it is much more sensitive to this treatment than the starter strains of *S. cerevisiae* frequently employed. In fact, growing the yeasts in rich medium, we found that a diploid laboratory strain of *S. cerevisiae* (HHD1; [39]) was inhibited by potassium bisulfite concentrations of 3 g/L; a commercial starter strain for vinification (Lalvin EC1118) still grew at more than 4 g/L, whereas the *H. uvarum* strain HHO1, from which all strains used in this work are derived, already ceased growth at less than 1.5 g/L.

To determine whether G6PDH contributes to sulfite resistance, similar halo assays to those described above for hydrogen peroxide were performed, with potassium bisulfite as an inhibitory agent at different concentrations (Figure 4b shows the results at 3.6 M). The sensitivity of the homozygous deletion strain (*Huzwf1Δ/Huzwf1Δ*) was clearly increased when compared to the wild-type control, and the heterozygous deletion still carrying one functional allele (*Huzwf1Δ/HuZWF1*) displayed an intermediary phenotype.

Since sulfite resistance may be of significant biotechnological importance for the future application of *H. uvarum*, this was further investigated. In wine strains of *S. cerevisiae*, sulfite resistance can be largely attributed to the overexpression of the *SSU1* gene, which encodes a sulfite pump [40]. Consequently, we placed this heterologous gene under the control of the strong *HuTEF1* promoter and stably integrated the construct at the *HuURA3* locus in strain HHO1, similar to the strategy explained in Figure 2b. As shown in Figure 4b (upper right corner and quantification), this strain (*Huura3::TEF1p-SSU1*) exhibited smaller halos of inhibition as compared to the wild-type control (*HuZWF1/HuZWF1*). This indicates that the heterologous *SSU1* gene is expressed and produces a functional sulfite exporter in *H. uvarum*.

## 3. Discussion

Glucose-6-phosphate dehydrogenase is an important enzyme not only for human health but also to ensure autonomous metabolism in microbial cells, including yeasts [1,2]. Here, we investigated the genetics and physiology of this enzyme in the abundant wine yeast *Hanseniaspora uvarum*, taking advantage of the recently developed methods for its genomic manipulation.

A homology search against the annotated *H. uvarum* genome sequence revealed the presence of only one G6PDH-encoding gene, consequently named *HuZWF1*. Besides the alignment of the deduced amino acid sequence with those of G6PDHs from humans and two other yeast species, revealing high sequence identities, structure predictions provided strong evidence for HuZwf1p being a functional homolog. Interestingly, HuZwf1p and its human homologue both carry an N-terminal extension, which is not present in the enzymes of the yeasts *Saccharomyces cerevisiae* or *Kluyveromyces lactis*. Although the extensions are different in their primary sequences, they both form an alpha-helical region according to the structure predictions. Apparently, this is not required for enzymatic catalysis, as the two other yeast enzymes, which do not carry the extension, confer G6PDH activity when expressed in a *Huzwf1* deletion strain. It is still possible that the extension could be important for fine-tuning enzyme activity and/or regulatory interaction with other proteins in *H. uvarum* and humans. While interesting, addressing this question would require elaborate and time-consuming experimental setups beyond the scope of this manuscript.

Given the strong similarities in the core enzyme structure and the fact that both human and yeast G6PDHs form homotetramers, we assume that HuZwf1p also adopts a tetrameric structure [28,41,42]. It yielded specific G6PDH activities in wild-type strains of *H. uvarum* of approximately 250 mU/mg protein. This is about 2.5-fold higher than the activity in a wild-type *S. cerevisiae* strain [19,43], indicating that *H. uvarum* has a higher capacity to channel glucose-6-phosphate into the pentose phosphate pathway. This relates to *H. uvarum* being a Crabtree-negative yeast, which relies on a more respiratory metabolism, as opposed to the mainly fermentative *S. cerevisiae*. Yet, the Crabtree-negative milk yeast *K. lactis* displays G6PDH activities of approximately 430 mU/mg protein, suggesting a still higher capacity [43]. In *K. lactis*, strong evidence for the PPP contributing substantially to glucose metabolism was provided by mutant analyses. Thus, the lack of activity of either a glycolytic enzyme or of transaldolase in the PPP did not preclude growth on glucose as a sole carbon source, whereas double mutants with blocks in both pathways were glucose-negative [26]. Similar experiments would be required in *H. uvarum* to demonstrate a significant contribution of the PPP to sugar metabolism. In fact, we are in the process of also constructing deletion mutants in the genes encoding both phosphofructokinase subunits, *HuPFK1* and *HuPFK2* ([33]; and unpublished results). However, since *H. uvarum* is diploid, with the necessity to delete two alleles of each gene, and lacks a sexual life cycle, impeding conventional crossing and tetrad analysis to combine genetic markers, construction of homozygous triple mutants may be hard to realize. This is further complicated by the expected glucose-negative phenotype of such triple mutants, which would require physiological studies to find a non-fermentable carbon source that supports the growth of *H. uvarum*.

Apart from these obstacles, the present work confirms previous findings that homologous recombination can be employed for efficient manipulation of *H. uvarum* [34,36]. Thus, all constructs for heterologous *ZWF1* gene expression integrated readily at the *HuURA3* locus, targeted by linearization of the vectors prior to transformation, as is common practice in *S. cerevisiae* [44]. Moreover, both *HuZWF1* alleles were deleted in one step from the diploid HHO44 genome by the use of a deletion cassette with long flanking homologies to the target sequence. This was not an isolated instance, as it also occurred in the deletion of one of the *HuPFK* genes (unpublished results from our laboratory). For future works, the investigation of a larger number of colonies arising on selective media in the first transformation may thus be worth the effort before applying the two-step deletion strategy to eliminate both target alleles, as suggested previously [36].

In the course of the heterologous *ZWF1* gene expression studies, we noticed that the genes from *S. cerevisiae* and *K. lactis* were very poorly expressed in *H. uvarum* under the control of their native promoters, reflected by the low specific G6PDH activities. This stands in contrast to the exchange of these and other genes between *S. cerevisiae* and *K. lactis*, which did not pose problems or require promoter modification (see, for example, [25,45] and references therein). However, it confirms previous suggestions that promoters from *S. cerevisiae* genes may not confer sufficient expression in *H. uvarum* [34]. Yet, when placed under the control of the strong native *HuTEF1* promoter, the heterologous genes were successfully expressed in *H. uvarum*. As a control, *HuZWF1* expressed from the strong promoter displayed almost three-fold higher activities than a wild-type strain. Although the two yeast enzymes and the *HuTEF1p-HsZWF1* construct conferred less activity (the latter at the lower end with approximately 100 mU/mg protein as compared to 250 mU/mg protein in wild-type *H. uvarum*), it would certainly allow further biochemical and biophysical protein characterizations. Yeasts clearly possess several advantages as heterologous expression hosts for human disease alleles: (i) They are eukaryotes and thus more suitable to produce human proteins than *E. coli*. (ii) With their short generation time, sufficient cell mass can be produced overnight for protein purifications. (iii) They can be easily genetically manipulated, as demonstrated herein. (iv) Most yeast species, including *H. uvarum*, are generally regarded as safe (GRAS). Regarding *H. uvarum* as an expression host, it should be noted that features like metabolic bottlenecks, the influence of protein folding, posttranslational modifications, or the burden generated by the overproduction of foreign proteins, as studied in the model yeast *S. cerevisiae*, still need to be thoroughly investigated. The current study only provides a first step in this direction.

The homozygous *Huzwf1* deletion mutant obtained was also subjected to phenotypic characterizations. As expected from data on *Sczwf1* mutants, the *H. uvarum* strain lacking G6PDH activity also required methionine for growth [15]. This has been attributed to a lack of NADPH, three molecules of which are required for reductive reactions in the biosynthesis of one molecule of methionine [13]. This feature of the *zwf1* deletion has been exploited for biotechnological purposes in *S. cerevisiae*, for instance, to improve xylose fermentation [46] or to construct a platform strain which overproduces tyrosine [47]. As methods for genetic manipulation of *H. uvarum* are only just emerging, and its ability to utilize pentoses as a sole carbon source has not been thoroughly investigated, our results on the role of G6PDH should be kept in mind until similar platform strains are available for this yeast. The *Huzwf1* mutant also showed a reduced growth on standard rich medium with glucose, a phenotype only observed in our laboratory strain of *S. cerevisiae* but not in others [15,18,36]. This indicates that the PPP indeed may contribute, to a larger extent, to sugar catabolism in *H. uvarum* as compared to *S. cerevisiae*.

A lack of G6PDH activity in *S. cerevisiae* has also been related to its oxidative stress response [13]. Accordingly, the first deletion mutant constructed proved to be hypersensitive towards hydrogen peroxide [15], a feature shared by strains of all genetic backgrounds tested so far [13,18,36]. It was thus not surprising to find an increased sensitivity towards this stress agent also for the homozygous *Huzwf1* deletion strain. This also makes sense in the natural environment of *H. uvarum*, as it frequently dominates the yeast population on grape skins [30]. The constant exposure to oxygen, in combination with its respiratory metabolism, is expected to produce reactive oxygen species as a side effect. Efficient detoxification mechanisms are thus a prerequisite. For instance, glutathione reductase is an enzyme for which the G6PDH reaction provides the reducing power as the primary source for NADPH in yeasts [48]. Moreover, glutathione synthesis itself is dependent on methionine metabolism, which, as outlined above, also requires G6PDH activity [49].

Finally, we observed that the homozygous *Huzwf1* deletion also results in an increased sensitivity towards sulfite as compared to wild-type *H. uvarum* strains. Although the *zwf1* deletions in other yeasts have not yet been tested for their effect on sulfite tolerance, a connection to oxidative stress response has been insinuated for *S. cerevisiae* [50]. It is likely that the molecular basis for this feature is the NADPH-dependent sulfite reductase encoded by *MET10* in *S. cerevisiae*, which is required for the reduction of sulfite as a precursor in methionine biosynthesis [51]. Clearly, sulfite resistance is of special interest for the biotechnological application of *H. uvarum* as the predominant yeast in the early stages of wine fermentations and would be a desirable trait. Thus, expanding the *S. cerevisiae* starter cultures in large-scale industrial winemaking by using mixed populations to improve wine quality, such as starter cultures of *S. cerevisiae* plus *H. uvarum* or other non-conventional yeasts, are under investigation [52,53,54,55]. As sulfite is commonly added for microbial stabilization and an antioxidant in winemaking, strains of *H. uvarum* with an increased tolerance would be advantageous. One would think that adaptive laboratory evolution (ALE) with stepwise exposure of a wine strain to increasing sulfite concentrations would be a reasonable approach [56]. However, sulfite resistance in wine strains of *S. cerevisiae* is mainly due to overexpression of the sulfite-pump encoding *SSU1* gene caused by chromosomal translocations [40]. In silico analyses revealed that the *H. uvarum* sequence probably lacks a homologue of this gene, diminishing the likelihood of an effective natural selection approach. Instead, our results suggest that heterologous expression of the *ScSSU1* gene in *H. uvarum* could be an alternative, as strains proved to be more resistant to sulfite than the recipient wild-type strain. Of course, this does not preclude the possibility of using in vitro mutagenesis on the *ScSSU1* expression vector, *SSU1* genes from other yeast species, and/or ALE on the manipulated *H. uvarum* strains to further enhance the resistance level. Also, it remains to be determined whether overexpression of *HuZWF1* in this context could also contribute to improving sulfite resistance.

In summary, we showed that the homozygous *Huzwf1* deletion strain can serve as a host for expression of heterologous *ZWF1* genes, including alleles with mutations from human patients. Although this has been previously exercised in a *S. cerevisiae* host for human G6PDH variants [57], *H. uvarum* may provide some advantages. For example, it grows faster and reaches higher cell densities than *S. cerevisiae*, providing more biomass for protein purification [33]. In addition, its respiratory metabolism provides a physiological environment resembling human cells more closely than the strongly fermentative baker’s yeast. The expression of the *ScSSU1* gene further demonstrated that heterologous expression is not restricted to G6PDH but can generally be applied to other proteins of interest. The genetic manipulations exercised herein will also increase the potential of *H. uvarum* for its different biotechnological applications [29].

## 4. Materials and Methods

### 4.1. Media, Strains, and Culture Conditions

Rich media were based on yeast extract (1% *w*/*v*) and peptone (2% *w*/*v*) from Difco Laboratories Inc., Detroit, MI, USA, with glucose (2% *w*/*v*) as a carbon source (YEPD). Synthetic media were based on Difco yeast nitrogen base with ammonium sulfate as described in [58], with the addition of amino acids and bases using a mixture provided by MP Biomedicals (Eschwege, Germany; CSM-His-Leu-Trp-Ura) supplemented as required for the selection of plasmids or deletion markers, and 2% glucose (*w*/*v*) as carbon source (SCD). In total, 400 mg/L of hygromycin B or 50 mg/L G418 were used for marker selection. For the preparation of solid media, 1.5% agar was added prior to sterilization. Yeasts were incubated at 28 °C, with constant agitation at 180 rpm for liquid cultures.

*H. uvarum* strains employed in this work are listed in Table 2. For the purpose of plasmid constructions by in vivo recombinations, the diploid *S. cerevisiae* strain DHD5 was used [58]. Comparative assays for sulfite resistance also included the S. cerevisiae strains HHD1 [39] and the commercial wine yeast strain Lalvin EC1118, kindly provided by Christian von Wallbrunn (Hochschule Geisenheim, Germany).

For work with *E. coli*, strain DH5α (Invitrogen, Karlsruhe, Germany) was employed, grown at 37 °C in LB medium (0.5% yeast extract, 1% Bacto peptone, 0.5% sodium chloride) and supplemented with ampicillin (50 mg/L) for plasmid selection.

### 4.2. Construction of Deletion Mutants and Cloning Procedures

For the transformation of *H. uvarum,* the freeze method was used as previously described [36], with the exception that cells were exposed to a heat shock for 5 min at 28 °C instead of 30 °C after the addition of DNA.

Deletions of the *HuZWF1* alleles were obtained by PCR-based one-step gene replacements. Oligonucleotides employed for this and other purposes are listed in Table 3.

To obtain longer flanking regions for homologous recombination in *H. uvarum*, the *HuZWF1* gene was first amplified by PCR with the primer pair 20.277/20.278, using DNA from strain HHO1 as a template and originally cloned as a BamHI/SalI fragment. Several steps of subcloning finally led to the insertion of the fragment into a derivative of the *S. cerevisiae* multicopy vector YEplac195 [59] to yield pJJH3286. The latter was linearized at the single BglII site, dephosphorylated, and cotransformed with PCR-generated deletion fragments into *S. cerevisiae* strain DHD5. Deletion cassettes were amplified with the primer pair 22.193/22.194 from pJJH3204 [36] to generate pJJH3287 (*Huzwf1::AgTEF2p-hph-CaURA3term*) and from pJJH1287 to generate pJJH3388 (*Huzwf1::AgTEF2p-KlLEU2-AgTEF2term*). Both PCR products included the flanking *loxP* sites. The insertions with the markers flanked by *HuZWF1* up- and downstream sequences were subcloned into pUK1921 [60], yielding pJJH3293 and pJJH3294, respectively, from which they were excised by digestion with BamHI/SphI prior to transformation of HHO44. As exercised previously for the construction of strains with auxotrophic markers [36], a consecutive strategy with the two selection markers was initially followed, resulting in strains with the genomic constitution depicted in Figure 2a and listed in Table 2. However, the approach of substituting *HuZWF1* by the *KlLEU2* marker from pJJH3394 already yielded two homozygous deletion clones (*Huzwf1::KlLEU2/Huzwf1::KlLEU2*) in the first attempt amongst several expected heterozygous ones (*Huzwf1::KlLEU2/HuZWF1*). After verification of the correct deletion by PCR with flanking and internal oligonucleotides, the episomal vector pJJH3203 (*kanMX HuTEF1p-Cre*) was introduced into one of the homozygous deletion strains to produce the Cre recombinase and remove both *KllEU2* marker alleles, retaining single *loxP* sites substituting the original *HuZWF1* open reading frames (Figure 2a; [36]). To induce plasmid loss, cells were grown overnight in non-selective rich medium, streaked out for single colonies on YEPD plates, and incubated for 2 days at 28 °C. They were then replica-plated onto YEPD with or without 50 mg/L of G418, and clones sensitive to the latter were picked for further investigations.

Integrative plasmids for expression of heterologous *ZWF1* genes were obtained by subcloning genes from various existing constructs [19], introducing new restriction sites by PCR if required. Clones containing PCR fragments were verified by Sanger sequencing (Seqlab, Göttingen, Germany) as not containing errors in the open reading frames before they were introduced into *H. uvarum* HHO44. Complete sequences of all plasmids described in the following are available upon request. As a vector for integration/disruption at the *HuURA3* locus, pJJH3313 was constructed (Figure 2b). It is based on the pUC19 backbone [61], into which the *HuSUI2p-hph-CaURA3term* region from pJJH3152 [36] was inserted, together with a polylinker to facilitate the cloning of heterologous genes. It also contains an internal fragment of the *HuURA3* gene (from position 112 to 470 in the open reading frame, amplified with the primer pair 21.304/21.305), which was cloned as an EcoRI/SalI fragment with a unique internal NheI site introduced for linearization to promote homologous recombination upon introduction into *H. uvarum*. This vector was used to insert different *ZWF1* genes as follows: The *ScZWF1* gene and the *KlZWF1* gene with their flanking regions were cloned as SphI/SalI fragments into pJJH3313 to yield pJJH3314 and pJJH3315, respectively. The SphI sites were introduced with the primers 23.089 and 23.090, respectively, in combination with 99.113 or 14.111 as required, amplifying the genes from clones previously obtained [19]. *HuZWF1* was cloned as a HindIII/SalI fragment, which also included the *hph* resistance cassette, to yield pJJH3320. The human G6PDH gene was first placed under the control of the strong *HuTEF1* promoter, which was introduced as an EcoRI/BamHI fragment into pJJH2223 [19] to yield pJJH3318. After further cloning steps, it was inserted as a NotI/SalI fragment into pJJH3313, resulting in pJJH3322 (Figure 2b). The latter was used as a basis to replace the *HsZWF1* coding sequence with those of the different yeast *ZWF1* genes. Thus, *ScZWF1* and *KlZWF1* were cloned as BamHI/XhoI fragments into pJJH3322 digested with BamHI/SalI to yield pJJH3350 and pJJH3351, respectively. Finally, *HuZWF1* was introduced into the pJJH3322 backbone as a BamHI/SalI fragment to give pJJH3352. All integrative plasmids were linearized with NheI and introduced into strain HHO1, selecting for hygromycin resistance. Correct insertions disrupting one of the *HuURA3* alleles were verified by PCR.

For heterologous expression of the *ScSSU1* gene, which encodes a sulfite pump, a similar strategy was followed. Thus, the *ScSSU1* sequence starting with the ATG translation start codon and carrying 345 base pairs of the terminator region was amplified by PCR from DNA of strain DHD5 with the primer pair 20.349/20.350. It was cloned into a *HuURA3* integration vector similar to pJJH3322 under the control of the *HuTEF1* promoter to yield pJJH3167. Integration was targeted by linearization with Bsp119I prior to the transformation of strain HHO1. Transformants were selected for hygromycin B resistance and verified for correct integration/disruption at the *HuURA3* locus.

### 4.3. Growth Curves

Cells were pregrown overnight in 3 mL of YEPD at 28 °C with shaking at 180 rpm, diluted to an OD_600_ of 0.3 in fresh SCD medium, and allowed to grow for another 5–6 h. They were adjusted to OD_600_ of 0.1 in SCD, and three technical replicates of each strain were applied to a microtiter plate. Growth curves were obtained in 100 µL cultures in 96-well plates in SCD medium and recorded in a Varioscan Lux plate reader (Thermo Scientific, Bremen, Germany). Except for the growth temperature of 28 °C, all settings and calculations were as previously described [62]. For statistical analysis and preparation of graphics, R v. 4.1.0 and R Studio v. 1.41717 were used [63]. The maximal growth rates were determined by moving a sliding window of width between 5 and 15 measurements along the dataset and sequentially performing linear regression.

### 4.4. Enzyme Assays

Cultures for enzymatic determinations were grown overnight in rich medium (YEPD), inoculated in fresh medium to an OD_600_ of 2.0, and grown for another 4–5 h with shaking at 180 rpm at 28 °C. Cells were then harvested by centrifugation, washed once with water and once with Tris-HCl buffer, pH7.5, and stored at −20 °C until use. Glucose-6-phosphate dehydrogenase activities were determined routinely from yeast crude extracts prepared by breaking of cells with glass beads and centrifugation, as described previously [19]. G6PD activities were determined in Tris-HCl buffer, pH7.5, by following the kinetics of NADP reduction at a final concentration of 0.4 mM, added from a 40 mM stock solution in extraction buffer, in a DU800 spectrophotometer (Beckman-Coulter, Krefeld, Germany) at 340 nm and 30 °C. Glucose-6-phosphate was added at a final concentration of 2 mM. All chemicals used for enzyme tests were obtained from Sigma-Aldrich Chemie GmbH, Munich, Germany.

### 4.5. Inhibition Halo Assays

To examine growth inhibition, strains were grown overnight in liquid-rich medium (YEPD), diluted to an OD_600_ of 1.0 (wild-type and heterozygous *Huzwf1Δ/HuZWF1* mutants) or 2.0–3.0 (homozygous *Huzwf1Δ/Huzwf1Δ* mutants), and 300 µL were used to cover SCD plates with 30 mL of medium lacking uracil with a homogenous lawn. After drying the plates in a sterile hood, circular filter papers soaked with the reagents (between 0–13.4 mM for hydrogen peroxide or 0–3.6 M for potassium bisulfite) were deposited.

Plates were incubated for 1–2 days at 28 °C, scanned, and the area of inhibition was determined using the Analyze Particles function of Fiji (based on ImageJ 153c (https://imagej.net/, accessed on 2 February 2024). For statistical analysis and preparation of graphics, R v. 4.1.0 and R Studio v. 1.41717 were used [63].

### 4.6. In Silico Analyses

The annotated *H. uvarum* genome sequence (GenBank accession number APLS01000000; [33]) was employed for homology searches and to extract the *HuZWF1* sequence. Alignments were performed with Clustal Omega [64]. For structure prediction of the HuZwf1p protein, the AlphaFold 2 program was used [37,38]. Calculated structures for G6PDH from the AlphaFold Database were used for *S. cerevisiae* and *H. sapiens*. The structure of the *Kluyveromyces lactis* G6PDH is an experimentally determined structure [42]. The Open-Source version of PyMOL v. 2.6.0a0 [65] was used for visualization and alignment of the protein structures.

## Figures and Tables

**Table 3:** Oligonucleotides used in this work.

Number	Name	Sequence (5′ → 3′) ^1^
20.277	HuZWF1forBam	CTTTGgatCCAGTAGTTGTGAATTATACGG
20.278	HuZWF1revSal	TATGTGTCGACCTTTTCAAGAGG
20.287	HuZWF1startBam	gcgtggatccATGTTTTTTAACGAAAAACGAATCGG
21.304	HuURA3intforEco	TGGAaTtcGTCAACACAACAGCTGAATTATTAG
21.305	HuURA3intforEco	ggctgtcGACCCTTTAGAACTTAATTCAG
22.193	Huzwf1del5	TTATTAGCGTTGTATATAACGACTTTGCTTGATTTTGTATGATGTTTGAATTTAT- GCTTCGTACGCTGCAGGTCGAC
22.194	Huzwf1del3	TTTAAAACCTTTTTTTTGATATAAAAACAATTGATATTTTATATATAATATGAAGCATAGGCCACTAGTGGATCTG
23.078	HuZWF1wfor	GGAACAAGAAAACAGTTCTTTAGAACTTG
23.079	HuZWF1wrev	GGCAGGTTCTATGCATGATTTGGATAG
23.080	HuZWF1for	GTGCCTGATGAGGAATCATCTTC
23.081	HuZWF1rev	CAAGAGGTTCCAAAATGGATATAC
23.089	ScZWF1forSph	gcgtgcatgCTGGTAAGTAAGGTGTAGTTTTG
23.090	KlZWF1forSph	gcgtgcatGCGATGAAAGCCAACAGTGGTTAG
23.143	HuZWF1forBam	gtgaggatccATGTTTTTTAACGAAAAACGAATCGG
20.349	ScSSU1BamATG	gcgtggatccATGGTTGCCAATTGGGTACTTG
20.350	ScSSU1revSal	ggctgtcgacGGTTGCAGAAAGCATAAAGTTCTG
99.113	universal-47	CGCCAGGGTTTTCCCAGTCACGAC
14.111	reverse-60	GCTTCCGGCTCGTATGTTGTGTGG
06.252	AgTEF2term3out	TCGCCTCGACATCATCTGCCCAG
10.225	AgTEF2prom5out	CCTCAGTGGCAAATCCTAAC
21.216	Hygro5out	GACAATTGCATCAAATCAGAAACAG
21.217	Hygro3out	GTTTTGGCTGATTCTGGTAATAGAAG

^1^ Sequences introduced for cloning purposes, which are not present in the template DNA, are highlighted in lowercase letters in red print. Red uppercase letters designate sequences binding to deletion cassettes. Blue letters indicate bases of restriction sites also present in the template sequence. General primers listed in the lower part were employed for cloning purposes and verification of deletions and integrations.

## Data Availability

Protein and nucleic acid sequences are available from the public databases as cited. Sequences of plasmids constructed and manipulated chromosomal loci are available upon request.

## Abbreviations

CFU = colony forming units; G6PDH = glucose-6-phosphate dehydrogenase; PPP = pentose phosphate pathway; ROS = reactive oxygen species; *ZWF* = gene encoding G6PDH (“Zwischenferment”).
